# Updated Meal Patterns in the Child and Adult Care Food Program and Changes in Quality of Food and Beverages Served: A Natural Experimental Study

**DOI:** 10.3390/nu14183786

**Published:** 2022-09-14

**Authors:** Tatiana Andreyeva, Rebecca S. Mozaffarian, Erica L. Kenney

**Affiliations:** 1 Department of Agricultural and Resource Economics, Rudd Center for Food Policy and Health, University of Connecticut, One Constitution Plaza, Hartford, CT 06103, USA; 2 Department of Nutrition, Harvard T.H. Chan School of Public Health, 655 Huntington Ave, Boston, MA 02115, USA; 3 Department of Social and Behavioral Sciences, Harvard T.H. Chan School of Public Health, 655 Huntington Ave, Boston, MA 02115, USA

**Keywords:** child nutrition, CACFP, menu analysis, preschools

## Abstract

With diet-related chronic diseases being the largest contributors to U.S. morbidity and mortality, identifying population-level strategies to promote healthier diets is essential. Intervention during early childhood may be particularly important. The Child and Adult Care Food Program (CACFP), a federal nutrition assistance program in the U.S. that supports serving meals and snacks in child care settings, reaches millions of U.S. children. Recent 2017 updates to CACFP’s meal patterns were meant to improve the nutritional quality of food served through CACFP by providing more whole grains, fruit, and vegetables. In this study, we used a natural experimental, longitudinal study of child care centers participating in CACFP compared to nonparticipating centers to assess whether the quality of food and beverages served (per menu analysis) improved following the CACFP meal pattern changes. While we found that CACFP centers were more likely to meet several key nutrition standards in comparison to non-CACFP centers overall, there were no differences in menu quality from before to after the 2017 standards change between CACFP and non-CACFP centers. Nutrition standards for CACFP may need to be further strengthened with adequate financial and technical support given to child care programs for effective implementation.

## 1. Introduction

Poor diet quality contributes more to global morbidity than any other behavioral, environmental, occupational, or metabolic risk factor [1]. Identifying how to shift dietary patterns towards choices that promote health and prevent chronic disease is a critical public health challenge. Ensuring that young children develop healthy eating habits may be a particularly effective strategy, given that habits formed during this developmental stage can persist throughout the life course [2]. However, in the U.S., young children currently consume foods high in sugar, sodium, and saturated fat and low in fiber, so diets of many children fall short of dietary recommendations [3,4].

Child care settings are important to focus on because they have the potential to help reshape food choices for the majority of American children who attend regular non-parental care, including center-based child care [5]. Children, especially those attending all day programs, could obtain a significant fraction of daily calories from meals and snacks served and have more choices in terms of a variety and types of foods served outside of home. Child care programs can influence children’s dietary intake by providing nutritious foods and beverages and implementing feeding practices that encourage healthy choices [6,7,8]. A large role in supporting nutrition in child care settings belongs to the USDA Child and Adult Care Food Program (CACFP), which serves 4.6 million children per year, targeting benefits to children from households with low incomes [9]. Foods provided to children in CACFP programs must meet specific nutrition standards in order to be reimbursed with federal funds. These standards can thus help ensure that the meals and snacks served to children in child care promote healthy eating habits and nutrition. 

Until recently, the nutrition standards for CACFP had not been updated to be in line with dietary science [10]. While there was some evidence that CACFP meals and snacks had some nutritional benefits over those served in non-CACFP participating programs, such as serving more fruit and vegetables and fewer sugary beverages, these benefits tended to be small and inconsistent across studies [11,12,13,14,15,16]. The standards themselves were originally designed before the onset of the childhood obesity epidemic and thus did not focus on nutrition for healthy child weight and chronic disease prevention [10]. In 2017, as a result of the Healthy, Hunger-Free Kids Act of 2010, the standards were updated for the first time since 1968 to be more in line with what dietary science has found promotes health and reduces risk of chronic disease [3]. The updated guidelines increase foods such as whole grain, fruit, and vegetable offerings and decrease added sugars [17]. Such changes could help promote healthier eating for the millions of mostly low-income children who attend CACFP-participating programs [9].

Emerging research suggests that the updated standards have been widely implemented [18] and may have resulted in some improvements in young children’s dietary intake [19,20]. However, studies to date have only examined changes within CACFP-participating programs or were limited to survey data only [21]. Without a comparison group of non-participating programs, it is difficult to assess whether any improvements in child care menu and/or meal quality are truly due to the updated meal pattern standards in CACFP or whether improvements may be due to natural time trends or some other influence on child care meals. Finally, prior research assessing menu quality in child care settings has been limited to cross-sectional studies and often lack comparison groups of non-participating programs [22,23].

This study aims to address this evidence gap by leveraging pre-update and post-update menu data on the reported meals and snacks served in a sample of both CACFP-participating and -nonparticipating child care centers. Using a longitudinal, difference-in-difference approach, this study evaluates the extent to which the 2017 CACFP meal pattern changes were associated with improvements in the quality of meals and snacks served. We hypothesized that: (1) CACFP participation would be associated with better menu quality as compared to nonparticipation both before and after the updates and (2) that menu quality would improve in CACFP-participating programs from before and after the updates while staying the same in nonparticipating programs.

## 2. Materials and Methods

### 2.1. Study Sample and Design

The sample consisted of licensed child care centers in the state of Connecticut (CT) that served meals and/or snacks to children 0–5 years of age (not including school-age after-school programs). To identify eligible centers, administrative records of licensed child care centers in the state were obtained in 2016 (prior to the implementation of the updated CACFP standards) and again in 2019 from the CT Office of Early Childhood. This list was compared to the State Department of Education’s records on CACFP participation. All CACFP-participating centers were assumed to serve meals and/or snacks by design. For non-participating centers, which could either opt to serve their own food or have parents/guardians send in meals and snacks, researchers verified via telephone whether the center served meals and/or snacks. The study invited all CACFP-participating centers (*n* = 176) and a sample of non-CACFP centers serving children ages 0–5 (*n* = 391 randomly selected from 733 non-CACFP centers serving young children and known to provide food) to participate. Non-CACFP centers located in low-income communities were oversampled to provide a stronger comparison group with CACFP centers given that CACFP participation is associated with serving low-income communities. 

To recruit these centers, center directors were contacted by email to participate in an online survey about food service practices affecting young children. This initial e-mail requested that the survey be completed by the person most familiar with the food service at the child care center. As part of the survey, participants were asked to submit a copy of their current menu to show food selections of children in their care. All participants consenting into the study and completing the survey received a monetary incentive (a USD 20 gift card). The survey data were reported elsewhere [13,21,24]. 

We collected data from 237 child care centers in 2016 as part of what was initially planned to be a cross-sectional study and then conducted a second data collection with a sample of 201 centers in 2019, many of whom had participated in the original data collection, after the updated CACFP standards had been implemented. Of these centers, directors provided weekly menus for 92 centers at both baseline and follow-up, which resulted in a final sample for longitudinal analysis of 55 non-CACFP participating centers and 37 centers participating in CACFP.

### 2.2. Measures

#### 2.2.1. Child Care Center-Level Characteristics

Child care providers completed surveys about center characteristics, including: CACFP participation status; whether the center was accredited by the National Association for the Education of Young Children (NAEYC); whether the center participates in Connecticut’s School Readiness program (which is an indicator of how many low-income children are served by the center); and non-profit/for-profit status. Providers also reported whether they had received CACFP training and what types of kitchen facilities their center had. Center capacity, i.e., the maximum number of enrolled children allowed per center, was determined from administrative licensing data, and data on household income, racial/ethnic composition, and poverty status for the U.S. Census block in which the center was located were assessed by linking the center’s address with data from the 2013–2017 American Community Survey [25].

#### 2.2.2. Menu Quality

For each participating child care center, the research team extracted one week’s worth of menu data. Each food and beverage item listed on the menu for each meal on the sampled week was classified into one or more food and beverage categories relevant to CACFP nutrition standards using a coding protocol from prior studies, which is available upon request [13,24,26]. We categorized milks as low-fat (1%) or skim, reduced fat (2%), whole fat, and flavored (for any flavor and percent fat). We classified other beverages as 100% fruit juice; fruit drinks/other sugar-sweetened beverages (SSBs, such as sodas, lemonades, non-100% juice drinks); or water (tap or bottled, unsweetened). We classified foods as follows: fruit of any type (excluding juice); vegetables of any type; dark green vegetables (e.g., spinach, broccoli, mixed greens); red and orange vegetables (e.g., carrots, squash, red peppers); starchy vegetables (e.g., potatoes, corn, green peas); legumes; grain products of all types; whole-grain products (first ingredient on nutrition label is a whole grain); refined grain products (first ingredient on package label is not a whole grain); grain products of unknown whole-grain content; grain-based desserts (cookies, pastries, granola bars); meat/meat alternates of any type (includes both meats and vegetarian protein sources); lean meats (poultry); red or processed meats (beef, lamb, goat, ham, sausage, hot dogs); pre-fried meats (chicken nuggets, chicken fingers, fish sticks); nuts/nut butter; eggs; natural cheese; processed cheese (American cheese, spray cheese, Velveeta); yogurt; tofu or other soy products; sweets (non-grain-based: ice cream, candy, pudding); and other foods. Cereals were evaluated for compliance with CACFP standards on sugar content by checking manufacturer’s website for nutrition information. 

With these coded data, we first assessed the extent to which center menus met basic CACFP standards for serving all required meal/snack components. Centers were coded as meeting basic component standards if they reported serving the following to preschool-age children: three required components for breakfast (fruits/vegetables, grain/meat/meat alternate, low-fat/skim milk); five required components for lunch (fruit, vegetable, grain, low-fat/skim milk, meat/meat alternate), and two components for snack (any of two of the five lunch components above) [9].

We then assessed if the food and beverage items on the menus met each of five of the 2017 updated daily minimum CACFP nutritional requirements for meals [17] served for each day in the sampled menu week. The five standards assessed were: (1) only unflavored low-fat/skim milk served to children ages 2–5 years old; (2) at least one serving of whole grains per day; (3) both fruit and vegetable served at lunch; (4) 100% fruit/vegetable juice limited to one serving per day; and (5) no grain-based desserts served as grains. We chose these five standards, as we were able to assess them with menu data; some other standards, such as eliminating on-site frying or limiting the sugar content of cereals and yogurts, were not possible to evaluate with menus at both time points. The minimum standard for serving fruit and vegetable at lunch was assessed per meal, and the other four minimum CACFP standards were assessed as meeting or not meeting the standards per day.

Lastly, we evaluated if menus met the following voluntary CACFP “best-practice” standards, which are encouraged but not required by CACFP: (1) serve fruit or vegetable as one of the two components at every snack; (2) serve whole fruit more often than juice; (3) serve dark green vegetables at least weekly; (4) serve red and orange vegetables at least weekly; (5) serve beans and legumes at least weekly; (6) serve starchy vegetables least weekly; (7) serve other vegetables at least weekly; (8) serve at least two servings of whole grains daily; (9) serve lean meats, nuts, and legumes only; (10) limit to one serving or less of processed meats weekly; (11) limit cheese to natural cheese only; (12) limit to one serving or less of pre-fried meat weekly; and (13) provide no non-creditable foods with added sugars (e.g., candy, sugary drinks). The best-practice standard for fruit or vegetable at snack was assessed per snack; whole fruit served more often than juice and at least two daily servings of whole grains were assessed per day; the remaining best-practice standards were assessed on a weekly basis. 

### 2.3. Statistical Analysis

We calculated the proportion of centers meeting each of the accreditations, type of preschool, staff CACFP training, meal preparation methods, access to food service facilities and equipment, and the mean (±SD) for descriptive and demographic data. Data were stratified by CACFP participation status and baseline (2016) versus follow-up (2019). Because many centers in our sample provided snacks but not lunches (these were household-provided) and thus were not comparable to centers providing lunches on several of the CACFP outcomes, we also stratified our analysis by centers that provided snacks only and centers that provided meals only or meals and snacks. 

We calculated the proportion of centers by CACFP status, year, and food service type that met each CACFP meal component requirement, minimum nutrition standard, and best-practice standard. We also calculated the mean (±SD) total number of minimum standards met per day and best-practice standards met each week. 

To test whether the updated minimum CACFP standards increased the nutritional quality of meals served in CACFP centers (based on menus), we used a difference-in-difference approach. Generalized estimating equations (GEE) logistic regression models were used to calculate the odds of meeting each of the CACFP minimum standards and voluntary best practices for menus, accounting for the clustering of menu observation days within centers. Models included a term for (1) time comparing 2019 to 2016; (2) CACFP status comparing CACFP to non-CACFP centers, and (3) an interaction term between time and CACFP status to estimate whether CACFP centers experienced additional changes in menu quality from 2016–2019 beyond the overall time effect (the difference-in-difference estimator and the CACFP policy change effect). To select demographic and center-characteristic covariates for the model, we used a backward selection process; the only covariate that meaningfully altered parameter estimates and remained significant was center capacity; thus, all models also adjusted for center capacity. Two-sided tests of significance were conducted in these models; alpha was set at *p* < 0.05.

The 2016 data collection was approved by the University of Connecticut Institutional Review Board in June 2015, and the 2019 study was approved in May 2018. Analyses were conducted on SAS 9.4. (Cary, NC, USA).

## 3. Results

The administrative and sociodemographic characteristics of the centers are presented in Table 1, showing important differences between CACFP- and non-CACFP participating programs. Of the 37 CACFP-participating centers in the final sample, 100% served meals and/or snacks, while only 18 (32.7%) of the 55 non-CACFP participating centers did; the remaining 37 non-CACFP centers served snacks only. All CACFP centers in the sample were accredited by NAEYC, while only 54.6% of non-CACFP centers were; similarly, participation in CT’s School Readiness program was much more common among CACFP centers. Capacity was higher on average in CACFP centers, and tuition was lower. CACFP centers were more likely to have kitchens on site or to heat up foods on site delivered from a vendor than non-CACFP centers, who were more likely to have partial kitchens or microwaves in classrooms. CACFP centers were located in areas that tended to have lower median household incomes and lower proportions of the population identifying as non-Hispanic White or as having a college degree and higher levels of poverty. 

The frequencies with which centers serving meals and snacks (*n* = 37 CACFP centers, *n* = 18 non-CACFP centers) met each of the minimum CACFP meal component and nutrition standards as well as the voluntary best-practice standards, are shown in Table 2; frequencies for centers only serving snacks are shown in Appendix A Table A1. At both time points, CACFP centers were significantly more likely to meet the minimum meal component requirements for breakfasts, lunches, and snacks than non-CACFP centers. They were also more likely to meet the 2017 updated minimum nutrition standards for serving both fruits and vegetables at lunch and serving at least one whole grain per day at both time points; both CACFP and non-CACFP centers were overwhelmingly likely, before and after the updates, to serve low-fat milk to 2–5-year-olds and to limit the serving of 100% juice to once per day. Meanwhile, CACFP and non-CACFP centers had similar frequencies of not serving grain-based desserts in 2016, and these frequencies both increased in 2019. In general, compliance with the voluntary best-practice standards was less prevalent among both CACFP and non-CACFP centers, with some exceptions. Particularly poor compliance was for serving only lean meats, nuts, and legumes for meat/meat alternates and serving natural cheese only. Although rates of serving at least two servings of whole grains improved over time, only one-third of non-CACFP centers and about one-half of CACFP centers managed to satisfy this standard in 2019. 

Results from the difference-in-difference GEE models are shown in Table 3 for centers serving meals and snacks (results for centers serving snacks only are shown in Appendix A Table A2). In evaluating the role of overall time trends and controlling for CACFP status, centers were more likely to limit grain-based desserts and serve whole fruits more often than juice in 2019 as compared to 2016 (aOR for grain-based dessert standard, 2019 vs. 2016: 4.40, 95% CI: 1.2, 16.2; aOR for whole fruit best-practice standard, 2019 vs. 2016: 4.23, 95% CI 1.5, 11.6); however, no other significant differences from 2016 to 2019 were observed. There were, however, several significant differences in the likelihood of meeting CACFP standards between non-CACFP and CACFP centers at baseline. In 2016, CACFP centers were more likely to serve both fruits and vegetables at lunch (aOR = 4.42, 95% CI 1.25, 15.5), serve whole grains at least once per day (aOR = 2.72, 95% CI: 1.3, 5.9), and not serve foods with added sugars (aOR = 3.50, 95% CI: 1.1, 11.3). However, when evaluating whether the change in CACFP standards in 2017 was associated with additional increases in meeting standards for CACFP centers, no significant changes at *p* < 0.05 were found.

## 4. Discussion

In this longitudinal, natural experimental study of whether the 2017 CACFP nutrition standard changes were associated with better adherence to best-practice standards for child care menus, we found that CACFP-participating centers had a higher likelihood of meeting several nutrition standards compared to non-participating centers. CACFP centers were overall more likely than non-CACFP centers to serve both a fruit and a vegetable at lunch, to serve whole grains at least once per day, to serve fruit more often than juice, and to refrain from serving foods with added sugars. However, at the same time, we also found that there was no evidence of an extra improvement in nutrition standard adherence for the CACFP centers related to the 2017 CACFP standard changes when comparing these centers to non-CACFP centers over time. 

Our findings regarding the higher likelihood of CACFP centers meeting several key nutrition standards (although not all) are similar to prior investigations comparing CACFP to non-CACFP centers using cross-sectional study designs [19,21]. Previous studies have also found that CACFP centers are more likely to serve whole grains and limit foods with added sugars such as sugary drinks and candy when compared to non-CACFP centers while also finding few consistent differences between CACFP and non-CACFP centers when it comes to the serving of fruit and vegetables and the types of meats/meat alternates served [11,13,14,15,21,24,27]. Menus from both CACFP participating and non-participating centers were highly adherent with the CACFP nutrition standards. The centers in this sample, regardless of CACFP participation, overwhelmingly served low-fat milk to 2–5-year-olds, offered fruits and vegetables daily, and limited serving juice and grain-based desserts, making it difficult to impossible for there to be a significant difference for CACFP centers. 

Previous evaluations of the 2017 CACFP nutrition standards change have examined changes among CACFP-participating programs only [18,19,20,26]. One study found significant increases in CACFP-participating center directors reporting not serving sugary cereals or flavored milk, serving 100% whole grain products, and serving processed meats less than once a week from before to after the change in standards [18]. Another found increases in the likelihood of meeting the whole-grain standard but no other significant changes [26], alongside findings that children increased intake of whole grains and fruit but did not increase intake of vegetables or milk [19]. Sisson et al. found that children’s intake of fiber increased and sugar decreased at CACFP centers from before to after the standards change, but that there were no significant changes in adherence to CACFP requirements and best practices [20]. Taken together with our study, which is the first to use a comparison group not exposed to CACFP to control for time effects independent of the standards change, these studies suggest that while there may have been some small improvements in what was served to and consumed by children in CACFP centers, this may not have been due to the change in standards themselves. 

The minimal impact of the standards change for CACFP can be contextualized by comparing with the impact of the standards changes for the National School Lunch and Breakfast Programs that were also required by the Healthy, Hunger-Free Kids Act of 2010 [28]. The USDA requested recommendations based on the best available science for both programs from the National Academies of Medicine (NAM) [10,29]. The recommendations for the school meals programs were nearly adopted in full by the USDA [30], and Congress authorized increases in school meal reimbursements to account for the higher food costs associated with compliance with these new standards as well as funding to support implementation [31]. In contrast, the final standards issued by USDA for CACFP were necessarily less of a change than what had been recommended by NAM because no corresponding increase in CACFP meal reimbursements was authorized, and no grants were set aside to support child care programs in updating kitchen equipment or training staff [17]. The differing strength in standards for school meals versus CACFP as well as the differing investment of resource appears to have resulted in differing impacts; while several studies have found the changes to school meals have significantly improved students’ nutritional quality [32,33] and reduced obesity risk [34,35], our study, alongside the prior evaluations within CACFP centers [19,20,26], suggests very limited impact. Although following the stronger voluntary best-practice standards for CACFP could potentially lead to larger impacts, there appears to be little indication that participating programs are aware of them. Training by CACFP state agencies to support adoption of the best practices may help. Resources for providing and encouraging such training by state CACFP and licensing agencies are needed.

**Strengths and Limitations**

Strengths of this study include the use of a longitudinal study design with a comparison group of centers not participating in CACFP. Nearly all studies of CACFP to date have used cross-sectional designs or have not leveraged a comparison group of nonparticipating centers, making it difficult to test whether any changes observed in CACFP menus over time are due to CACFP’s nutrition standards changes or whether they might be due to an underlying trend affecting all centers regardless of CACFP participation. An additional strength was the use of centers’ menus for analyzing nutrition standard adherence rather than relying on self-report measures, which may not be accurate. 

A limitation of this study is the unbalanced covariates between the CACFP and non-CACFP centers. Because this study could not use a randomized, controlled trial design, we made every effort to draw a comparison sample of non-participating (i.e., unexposed to the nutrition standards changes) centers that would be as similar to CACFP centers as possible, limiting the sample to only those providing meals and attempting to oversample from low-income areas. Despite these efforts, the nonparticipating centers were substantially different from the CACFP centers. They were less likely to be accredited, to serve children from households with low incomes, and to have kitchen facilities while having higher tuition. Such differences may be impossible to avoid, given that CACFP is uniquely focused on centers that serve children from households with low income and centers make a choice to join the program. Despite these differences, our analyses indicated that these covariates were not associated with menu quality, suggesting the differences were unlikely to be confounders. However, the possibility of confounding cannot be ruled out. Another important limitation of the longitudinal menu assessment was a lower response rate than in cross-sectional surveys [21,24].

This study also was not able to evaluate whether there were differences in children’s actual consumption of foods and beverages served over time. Our study does not evaluate what foods and beverages were served to children or consumed by children. While evaluating menus and their compliance with nutrition standards is important, data on meals served and consumed are needed and should be prioritized in future studies. Such studies can answer a critical question of nutrient density, which menu assessments cannot. Finally, our analysis was limited to one state (Connecticut), which could affect generalizability of our findings, particularly with respect to states with much higher CACFP participation rates. Future research should prioritize using national longitudinal samples to understand impacts of the CACFP meal patterns across diverse areas and child care programs. While nationally representative studies examining CACFP exist [16], they are not currently longitudinal and do not involve examination of meals for unexposed children in non-CACFP programs. 

## 5. Conclusions

Participation in CACFP is associated with increased likelihood of serving whole grains, fruits and vegetables instead of juice, and refraining from serving foods and beverages with added sugars. However, the updates to CACFP’s nutrition standards via the Healthy, Hunger-Free Kids Act of 2010 were not associated with improved likelihood of centers adhering to nutrition standards when accounting for changes that may have been occurring over time for centers regardless of CACFP participation. To effect more beneficial changes to the foods and beverages served to the millions of children who receive subsidized meals through CACFP, stronger nutrition standards for the program, with appropriate accompanying financial and technical support for implementation, may be needed. 

## Figures and Tables

**Table 1:** Demographics characteristics of *n* = 92 Connecticut-licensed child care centers in 2019 ^1^.

	Non-CACFP Centers (*n* = 55)	CACFP Centers (*n* = 37)	*p*-value ^3^
*Meal and/or snack served, n (%):*			<0.001
Meal only or meal and snack served	18 (32.7)	37 (100)	
Snack only served	37 (67.3)	0 (0.0)	
*Accreditations, n (%):*			
Center accredited by the National Association for the Education of Young Children (NAEYC)	30 (54.6)	37 (100)	<0.001
Center has School Readiness program slots available	8 (15.1)	30 (81.1)	<0.001
Center has a sponsoring organization, n (%) ^1^	12 (21.8)	13 (39.4)	0.08
Center capacity, mean (SD)	88.4 (60.1)	117.9 (94.3)	0.10
Weekly tuition, mean (SD) ^2^	USD 262.4 (78.7)	USD 210.2 (57.9)	0.003
*Meal preparation, n (%):*			<0.001
Kitchen on site from scratch	11 (20.0)	19 (51.4)	
Kitchen of another center and delivered	0 (100)	0 (100)	
Purchased from a vendor and heat up on site	5 (9.1)	5 (13.5)	
Purchased from a vendor and do not heat up	8 (14.6)	7 (18.9)	
Other	31 (56.4)	6 (16.2)	
*Kitchen and equipment, n (%):*			0.003
Full kitchen on site	25 (45.5)	24 (62.2)	
Partial kitchen on site (fridge/freezer alone)	21 (38.2)	10 (27.0)	
Microwaves in the classroom	15 (27.3)	1 (2.7)	
No cooking equipment on site	4 (7.3)	1 (2.7)	
*Sociodemographic characteristics of center’s Census tract, mean (SD):*			
Median household income	USD 98,717 (USD 43,852)	USD 52,255 (USD 24,791)	<0.001
Percent of population with college degree	47.1 (16.4)	28.6 (21.4)	<0.001
Percent of households below poverty level	6.4 (6.2)	21.2 (13.7)	<0.001
Percent of population that is non-Hispanic White	78.8 (12.9)	47.2 (28.9)	<0.001

Notes: ^1^ Center characteristics were also measured in 2016; as these variables did not differ significantly in 2019, we present 2019 values only for clarity, but 2016 data are available on request; ^2^ measured in 2016 data collection; ^3^ *p*-values are from chi-square or Fisher’s exact tests for categorical variables and *t*-tests for continuous variables.

**Table 2:** Frequencies of menus meeting CACFP minimum standards and voluntary best practices among non-CACFP and CACFP centers providing meals and snacks in Connecticut, 2016–2019.

	2016 (Old CACFP Meal Pattern)		2019 (New CACFP Meal Pattern)	
	Non-CACFP Centers	CACFP Centers	Non-CACFP Centers	CACFP Centers
N menu days	90	179	89	185
N meals and snacks	246	492	232	544
Breakfast, n (%)	35 (14.2)	159 (32.3)	54 (23.3)	175 (32.2)
Lunch, n (%)	86 (35.0)	174 (35.4)	80 (34.5)	180 (33.1)
Snack (a.m./p.m.), n (%)	125 (50.8)	159 (32.3)	98 (42.2)	189 (34.7)
**Meeting CACFP meal component requirements, n (%) of meals ^1^**				
Breakfasts	30 (85.7)	153 (96.2)	40 (74.1)	159 (90.9)
Lunches	57 (66.3)	149 (85.6)	50 (62.5)	163 (90.6)
Snacks	112 (89.6)	154 (96.8)	81 (82.7)	184 (97.4)
**Meeting CACFP minimum nutrition standards, n (%) of days**				
Fruit and vegetables as 2 components at lunch	66 (76.7)	161 (92.5)	64 (80.0)	164 (91.1)
Unflavored skim/low-fat milk to 2–5-year-old children	84 (93.3)	178 (99.4)	84 (94.4)	183 (98.9)
≥1 serving of whole grains per day	35 (38.9)	110 (61.5)	49 (55.1)	160 (86.5)
Limit 100% fruit/vegetable juice to 1 serving per day	84 (93.3)	173 (96.7)	89 (100)	184 (99.5)
No grain-based desserts served	78 (86.7)	150 (83.8)	86 (96.6)	177 (95.7)
*Mean (SD) total number of minimum standards met per day*	4.1 (0.8)	4.4 (0.7)	4.4 (0.7)	4.9 (0.6)
**Following CACFP voluntary best practices, n (%) of snacks, days, and weeks**				
*Meal-level best practices*				
Fruit or vegetable as 1 of 2 components at snack	93 (74.4)	95 (61.6)	61 (62.2)	107 (56.6)
*Daily best practices*				
Whole fruit served more often than juice	59 (65.6)	156 (87.2)	78 (87.6)	168 (90.8)
≥2 servings of whole grains per day	13 (14.4)	50 (27.9)	27 (30.3)	101 (54.6)
*Weekly best practices*				
Dark green vegetables ≥ 1 time/week	11 (61.1)	25 (69.4)	10 (55.6)	27 (73.0)
Red and orange vegetables ≥ 1 time/week	14 (77.8)	33 (91.7)	15 (83.3)	32 (86.5)
Bean and peas ≥ 1 time/week	11 (61.1)	13 (36.1)	10 (55.6)	20 (54.1)
Starchy vegetables ≥ 1 time/week	13 (72.2)	27 (75.0)	14 (77.8)	29 (78.4)
Other vegetables ≥ 1 time/week	18 (100)	34 (94.4)	17 (94.4)	36 (97.3)
Processed meats ≤ 1 time/week	15 (83.3)	28 (77.8)	15 (83.3)	37 (100)
Pre-fried meats ≤ 1 time/week	18 (100)	28 (77.8)	15 (83.3)	36 (97.3)
Natural cheese only	0 (0.0)	0 (0.0)	1 (5.9)	3 (8.6)
Only lean meats, nuts, and legumes served for meat/meat alternates	4 (22.2)	3 (8.3)	3 (17.7)	3 (8.3)
No non-creditable foods with added sugars served	7 (38.9)	25 (69.4)	10 (55.6)	23 (62.2)
*Mean (SD) total number of best practices followed per week*	7.3 (1.6)	6.9 (1.5)	7.2 (1.5)	7.6 (1.5)

Notes: ^1^ Meal component requirements for CACFP are as follows: Breakfast (three components): milk, vegetables, fruit, or both and grains; Lunch (five components): milk, meat/meat alternates, vegetables*, fruit, and grains; Snacks (two components from milk, meat/meat alternates, vegetables, fruit, grains). *2 servings of vegetables were not considered due to low availability of this practice.

**Table 3:** Difference-in-difference model results: centers servings meals or meals and snacks.

	2019 (Post-Update) vs. 2016 (Pre-Update)			CACFP vs. Non CACFP			Interaction between 2019 to 2016 and CACFP Status		
	Odds Ratio	95% CI	*p*-Value	Odds Ratio	95% CI	*p*-Value	Odds Ratio	95% CI	*p*-Value
*CACFP minimum nutrition standards*									
Fruit and vegetables as 2 components at lunch	1.09	0.38, 3.1	0.88	4.42	1.25, 15.5	0.02	0.78	0.20, 3.04	0.72
Unflavored skim/low fat milk to 2–5-year-old children	0.76	0.13, 4.3	0.75	13.0	0.98 172.2	0.05	0.66	0.12, 3.71	0.64
At least one serving of whole grains per day	1.97	0.98, 4.0	0.06	2.72	1.3, 5.9	0.01	2.02	0.75, 5.4	0.17
No grain-based dessert	4.40	1.2, 16.2	0.03	0.80	0.42, 1.5	0.49	0.97	0.22, 4.4	0.97
*Best Practice Standards*									
Fruit or vegetable as 1 of 2 components at snack	0.41	0.12, 1.46	0.17	0.19	0.08, 0.50	<0.001	2.27	0.59, 8.82	0.23
Whole fruit served more often than juice	4.23	1.5, 11.6	0.005	4.82	1.4, 16.2	0.01	0.34	0.10, 1.15	0.08
At least 2 servings of whole grains per day	2.77	1.0, 7.7	0.05	2.53	0.80, 8.0	0.11	1.14	0.35, 3.71	0.82
Dark green vegetables at least once per week	0.79	0.27, 2.3	0.66	1.58	0.47, 5.27	0.46	1.5	0.38, 6.0	0.56
Red and orange vegetables at least once per week	1.50	0.29, 7.9	0.63	4.33	0.82, 22.8	0.08	0.97	0.04, 3.29	0.38
Bean and peas at least once per week	0.77	0.32, 1.83	0.55	0.36	0.11, 1.2	0.10	2.97	0.92, 9.6	0.07
Starchy vegetables at least once per week	1.50	0.64, 3.5	0.35	1.26	0.36, 4.4	0.72	0.80	0.21, 3.0	0.73
Lean meats, nuts, and legumes only	0.71	0.20, 2.5	0.60	0.31	0.06, 1.6	0.16	1.41	0.27, 7.2	0.68
No non-creditable foods with added sugars, including candy, sugary drinks	1.99	0.61, 6.5	0.26	3.50	1.1, 11.3	0.04	0.36	0.09, 1.4	0.14

Notes: All regressions adjusted for center capacity. The CACFP 2017 minimum nutrition standard of limiting 100% fruit/vegetable juice to one serving per day as well as the CACFP best-practice standards of serving other vegetables at least once per week, serving processed meats once a week or less, serving pre-fried meats once a week or less, and serving only natural cheese were not modeled due to lack of non-positive cases in certain cells.

## Appendix A

**Table A1:** Frequencies of meeting CACFP minimum standards and voluntary best practices among non-CACFP and CACFP centers providing snacks only, Connecticut, 2016–2019.

		2016 (Pre-Update)		2019 (Post-Update)	
		Non-CACFP Centers	CACFP Centers	Non-CACFP Centers	CACFP Centers
N days		254	163	247	174
N snacks		449	169	408	189
Morning snack, n (%)		234 (52.1)	11 (6.5)	227 (55.6)	15 (7.9)
Afternoon snack, n (%)		215 (47.9)	158 (93.5)	181 (44.4)	174 (92.1)
N weeks		51	33	50	35
*CACFP meal component requirements, n (%)*					
Morning snack		198 (84.6)	10 (90.9)	202 (89.0)	15 (100)
Afternoon snack		175 (81.4)	153 (96.8)	161 (89.0)	169 (97.1)
*CACFP minimum nutrition standards, n (%)*					
N days		254	163	247	174
Unflavored skim/low-fat milk to 2–5-year-old children		253 (99.6)	162 (99.4)	242 (98.0)	172 (98.9)
At least one serving of whole grains per day		76 (29.9)	27 (16.6)	67 (27.3)	58 (33.3)
Limit 100% fruit/vegetable juice to one serving per day		237 (93.3)	162 (99.4)	233 (94.3)	174 (100)
No grain-based dessert		220 (86.6)	133 (81.6)	224 (90.7)	166 (95.4)
*Mean total number of minimum standards met per day, SD*		3.1 (0.7)	3.0 (0.6)	3.1 (0.7)	3.3 (0.6)
*CACFP best practices, n (%)*					
Morning snacks, n		234	11	227	15
Fruit or vegetable as 1 of 2 components, n (%)		139 (59.4)	7 (63.6)	140 (61.7)	11 (73.3)
Afternoon snacks, n		215	158	181	174
Fruit or vegetable as 1 of 2 components, n (%)		149 (69.3)	96 (60.8)	120 (66.3)	96 (55.2)
Day service, n		254	163	247	174
Whole fruit served more often than juice, n (%)	106 (41.7)		59 (36.2)	118 (47.8)	72 (41.4)
≥2 servings of whole grains per day, n (%)	7 (2.8)		2 (1.2)	6 (2.4)	1 (0.6)
**Weeks, n**	51		33	50	35
Dark green vegetables at least once per week, n (%)	3 (5.9)		1 (3.0)	3 (6.0)	1 (2.9)
Red and orange, n (%) vegetables at least once per week, n (%)	19 (37.3)		4 (12.1)	13 (26.0)	5 (14.3)
Bean and peas at least once per week, n (%)	17 (33.3)		5 (15.2)	17 (34.0)	6 (17.1)
Starchy vegetables at least once per week, n (%)	3 (5.9)		0 (0.0)	1 (2.0)	1 (2.9)
Other vegetables at least once per week, n (%)	20 (39.2)		10 (30.3)	25 (50.0)	14 (40.0)
Processed meats one serving per week or less, n (%)	51 (100)		33 (100)	50 (100)	35 (100)
Pre-fried meats one serving per week or less, n (%)	51 (100)		33 (100)	50 (100)	35 (100)
Natural cheese only, n (%)	4 (11.8)		5 (21.7)	13 (44.8)	13 (50.0)
Lean meats, nuts, and legumes only, n (%)	21 (95.5)		11 (100)	21 (95.5)	10 (100)
No non-creditable foods with added sugars, including candy, sugary drinks, n (%)	30 (58.8)		32 (97.0)	38 (76.0)	33 (94.3)
*Mean total number of best practices met per week, SD*	4.7 (2.1)		4.3 (1.5)	5.1 (1.6)	4.5 (1.4)

Notes: Meal component requirements for CACFP are as follows: Snacks (two components from milk, meat/meat alternates, vegetables, fruit, grains).

**Table A2:** Difference-in-difference model results: centers servings snacks only.

		2016 (Pre-Update) vs. 2019 (Post-Update)			CACFP vs. Non CACFP			Interaction between 2019 to 2016 and CACFP Status		
	n	Odds Ratio	95% CI	*p*-Value	Odds Ratio	95% CI	*p*-Value	Odds Ratio	95% CI	*p*-Value
*CACFP minimum nutrition standards*										
Unflavored skim/low-fat milk to 2–5-year-old children	838	0.16	0.005, 5.2	0.30	2.40	0.0005, 11,895	0.84	3.06	0.11, 86.5	0.51
At least one serving of whole grains per day	838	0.87	0.58, 1.3	0.52	0.49	0.29, 0.85	0.01	2.62	1.2, 5.5	0.01
No grain-based dessert	838	1.47	0.87, 2.5	0.15	0.69	0.43, 1.1	0.13	3.12	1.2, 7.9	0.02
*Best practice standards*										
Morning snack: Fruit or vegetable as 1 of 2 components	487	1.11	0.72, 1.70	0.65	1.02	0.19, 5.6	0.98	1.39	0.16, 12.1	0.76
Afternoon snack: Fruit or vegetable as 1 of 2 components	728	0.86	0.58, 1.3	0.48	0.70	0.40, 1.2	0.20	0.91	0.50, 1.6	0.75
Whole fruit served more often than juice	838	1.24	0.84, 1.8	0.28	0.81	0.46, 1.4	0.46	0.91	0.49, 1.7	0.77
At least 2 servings of whole grains per day	838	0.87	0.29, 2.6	0.80	0.38	0.07, 2.0	0.26	0.54	0.03, 8.9	0.66
Dark green vegetables at least once per week	169	1.01	0.23, 4.5	0.99	0.40	0.02, 7.4	0.54	0.97	0.22, 4.3	0.94
Red and orange vegetables at least once per week	169	0.59	0.27, 1.3	0.18	0.23	0.07, 0.77	0.02	2.00	0.43, 9.2	0.38
Bean and peas at least once per week	169	0.98	0.51, 1.9	0.96	0.30	0.10, 0.94	0.04	1.23	0.36, 4.2	0.74
Other vegetables at least once per week	169	1.51	0.90, 2.5	0.12	0.68	0.26, 1.7	0.42	0.93	0.29, 3.0	0.90
Natural cheese only	169	6.10	1.6, 22.6	0.007	2.07	0.49, 8.8	0.32	0.60	0.10, 3.6	0.58
No non-creditable foods with added sugars, including candy, sugary drinks	169	2.13	1.0, 4.3	0.04	30.5	2.6, 352.9	0.006	0.23	0.01, 3.9	0.31

Notes: All regressions adjusted for center capacity. The CACFP 2017 minimum nutrition standard of limiting 100% fruit/vegetable juice to one serving per day as well as the CACFP best practice standards of serving starchy vegetables at least once per week, serving processed meats once a week or less, serving pre-fried meats once a week or less, and serving lean meats, nuts, and legumes only were not modeled due to lack of non-positive cases in certain cells.
